# 血液病患者合并肝脾念珠菌病的临床特征及预后

**DOI:** 10.3760/cma.j.cn121090-20231230-00347

**Published:** 2024-07

**Authors:** 丹苹 朱, 瑞 马, 云 何, 雪宜 罗, 伟 韩, 川 李, 婧睿 周, 益 廖, 博瑞 唐, 龙通 龙卡, 晓军 黄, 于谦 孙

**Affiliations:** 北京大学人民医院，北京大学血液病研究所，国家血液系统疾病临床医学研究中心，造血干细胞移植治疗血液病北京市重点实验室，北京 100044

## Abstract

肝脾念珠菌病是一种罕见的念珠菌病类型，可发生在血液系统恶性肿瘤、造血干细胞移植患者中，然而目前仍缺乏中国血液病合并肝脾念珠菌病患者的相关研究。本研究依据2020年《血液病/恶性肿瘤患者侵袭性真菌病的诊断标准与治疗原则（第六次修订版）》的诊断标准，回顾性分析了2008年至2022年在北京大学血液病研究所接受治疗并合并肝脾念珠菌病患者的临床特点及预后。研究最终共有18例患者诊断为肝脾念珠菌病，包括确诊1例（5.6％）、临床诊断2例（11.1％）、拟诊15例（83.3％）。其中3例（16.7％）患者发生在单倍体造血干细胞移植后，15例（83.3％）患者发生在化疗后。6例（33.3％）患者血培养阳性，其中热带念珠菌4例，白念珠菌2例。抗真菌治疗4周时，10例（58.8％）患者达部分缓解；8周时1例（6.3％）达完全缓解，10例（62.5％）达部分缓解。诊断后6个月时，3例（16.7％）患者死于血液学复发，没有患者死于肝脾念珠菌病。肝脾念珠菌病作为一种罕见的真菌感染性疾病，具有微生物学检查和组织学检查阳性率较低、治疗周期长、不易缓解等特点，在伴有持续发热的血液病患者中需警惕肝脾念珠菌病的发生。

## Abstract (en)

Hepatosplenic candidiasis（HSC）is a rare type of candidiasis that can occur in patients with hematologic malignancies, hematopoietic stem cell transplantation. At present, there is still a lack of studies on HSC in patients with hematologic disorders. Based on The Chinese Guidelines for the Diagnosis and Treatment of Invasive Fungal Disease in Patients with Hematological Disorders and Cancers（the 6th revision）, We retrospectively analyzed the clinical characteristics and prognosis of patients with HSC treated in Peking University Institute of Hematology from 2008 to 2022. Finally, eighteen patients were included, with 1（5.6％）proven, 2（11.1％）probable, and 15（83.3％）possible HSC. Among them, 3（16.7％）patients occurred after haploid hematopoietic stem cell transplantation and 15（83.3％）patients occurred after chemotherapy. 6（33.3％）patients had positive blood cultures, including 4 cases of Candida tropicalis and 2 cases of Candida albicans. At 4 weeks of antifungal therapy, 10（58.8％）patients achieved partial response（PR）, At 8 weeks, 1（6.3％）patients achieved complete response and 10（62.5％）patients achieved PR. At 6 months after diagnosis, 3（16.7％）patients died of hematopoietic recurrence, and none of them died of HSC. As a rare fungal infection disease, HSC has a low positive rate of microbiological and histological examinations, a persistent treat cycle, and has difficulty in remission, reminding us of the need for vigilance in patients with hematopoietic disorders and persistent fever.

侵袭性念珠菌病（IC）是血液病患者第二常见的侵袭性真菌病（IFD）类型，占所有IFD的25％～30％[1]–[3]。其发生的机制主要是各种原因导致的皮肤黏膜屏障受损后，定植在皮肤黏膜表面的念珠菌侵入血流，形成念珠菌血症，严重者可播散至器官形成播散性念珠菌病。播散性念珠菌病根据临床表现不同而分为急性和慢性。慢性播散性念珠菌病（CDC）是IC的一种独特表现形式，主要累及肝脏和脾脏，偶可累及肾脏等其他器官，故又称为肝脾念珠菌病（HSC）[4]。HSC通常发生在免疫功能低下的患者中，如血液系统恶性肿瘤化疗患者、造血干细胞移植患者以及中性粒细胞缺乏（粒缺）症患者的粒细胞缺乏恢复期[1],[4]–[6]。HSC的临床特征不具有特异性，确定诊断较为困难，目前的诊断主要依赖于患者的临床特征、微生物学检查和影像学检查；当血液病患者合并持续的发热、腹痛、肝脏和（或）脾脏肿大[7]表现时，需引起临床医师关注。此外，HSC的治疗通常具有周期长、不易缓解的特点[8]，持续性抗真菌药物的使用降低了患者的依从性，增加了患者的脏器毒性。HSC发病率较低，文献报道较少，国内更是缺乏HSC相关临床特点的报道。本研究旨在回顾性分析血液病患者合并HSC的临床特征及预后，为临床HSC的诊断、治疗及预防提供参考。

## 病例与方法

一、病例

本研究回顾性分析了2008年至2022年在北京大学人民医院被诊断HSC的血液系统恶性肿瘤患者。HSC诊断根据2019年欧洲癌症研究和治疗组织-感染性疾病协作组（EORTC-IDG）和美国真菌病研究组（MSG）标准、2020年《血液病/恶性肿瘤患者侵袭性真菌病的诊断标准与治疗原则（第六次修订版）》定义[9]–[10]。确诊HSC定义为在非黏膜组织采用针吸或活检取得的标本，通过组织化学或细胞化学的方法检出酵母菌细胞或假菌丝，或通常无菌而临床表现或放射学检查支持存在感染部位（不包括尿道、副鼻窦和黏膜组织），无菌术下取得的标本培养结果呈阳性；临床诊断HSC定义为患者同时满足宿主因素、临床标准［此前2周内出现念珠菌血症，且肝脏和（或）脾脏具有典型“牛眼征”病变的影像学表现］以及真菌学证据；拟诊HSC定义为患者存在宿主相关危险因素并且影像学检查显示肝脏或脾脏靶点样脓肿改变。

本研究为回顾性观察性研究，利用北京大学人民医院大数据平台、北京大学人民医院数据管理系统进行资料收集，在数据收集过程中充分保护患者隐私，符合免除伦理申请的条件。患者随访时间为诊断日期至诊断后4周、8周，患者至少随访6个月。

二、定义

粒缺定义为中性粒细胞绝对计数（ANC）<0.5×10^9^/L。对于随访时的疗效评估，完全缓解定义为患者在观察期内存活，HSC相关症状和体征、影像学异常全部消失，微生物学证据提示真菌清除，对于IC患者要求原感染部位再次或反复活检、真菌培养阴性；部分缓解定义为患者在观察期内存活，IFD相关症状和体征、影像学异常有所改善，微生物学证据提示真菌清除，对于IC或念珠菌血症患者需再次或反复活检、真菌培养阴性，HSC患者应退热且影像学稳定；疾病稳定定义为患者在观察期内存活，IFD相关症状和体征无改善，且临床、影像学、微生物学综合评估未提示疾病进展，对于IC患者持续可从血液或其他无菌标本中分离出念珠菌；疾病进展定义为临床、影像学和微生物学综合评估提示疾病进展，包括临床症状及体征加重或恶化（如感染性休克、播散性真菌病），影像学出现新发病灶或原发病灶的加重或扩大，可持续分离出真菌或活检阳性；疾病死亡定义为与HSC直接或间接相关的各种原因导致的死亡[10]。

三、统计学处理

本研究为描述性分析，采用SPSS 25.0进行统计学描述。连续变量采用均数±标准差表示（数据符合正态分布）或中位数（范围）表示（数据不符合正态分布），分类变量采用例数（构成比）表示。

## 结果

一、患者基本特征

共18例患者诊断为HSC，基本特征见表1。其中3例（16.7％）患者发生在单倍型造血干细胞移植后，15例（83.3％）患者发生在化疗后。在18例患者中，14例（77.8％）进行了抗真菌预防治疗（其中唑类药物占85.7％）。6例患者有既往真菌感染史，其中确诊2例（33.3％）、拟诊3例（50％）、未确定1例（16.7％）；2例（33.3％）真菌分别为热带念珠菌、近平滑念珠菌，其余4例（66.7％）真菌种类未知。

**表1:** 18例肝脾念珠菌病（HSC）患者的基本特征

基本特征	数值
男性[例（%）]	13（72.2）
年龄[岁，*M*（范围）]	36（4~58）
基础血液肿瘤[例（%）]	
急性髓系白血病	11（61.1）
急性淋巴细胞白血病	6（33.3）
急性双表型白血病	1（5.6）
原发病治疗方式[例（%）]	
化疗	15（83.3）
造血干细胞移植	3（16.7）
HSC诊断时危险因素[例（%）]	
黏膜损伤	4（22.2）
中心静脉置管^a^	12（80.0）
ICU收治	1（5.6）
胃肠外营养	5（27.8）
CMV血症	4（22.2）
EBV血症	1（5.6）
真菌血培养阳性[例（%）]	
热带念珠菌	4（22.2）
白色念珠菌	2（11.1）
肝脏活检[例（%）]^b^	
热带念珠菌	1（16.7）
未知病原体	1（16.7）
阴性	4（66.7）
G试验阳性[例（%）]^c^	6（42.9）
EORTC/MSG级别[例（%）]	
确诊	1（5.6）
临床诊断	2（11.1）
拟诊	15（83.3）
抗真菌预防治疗[例（%）]^d^	
氟康唑	1（7.1）
伏立康唑	8（57.2）
伊曲康唑	3（21.4）
卡泊芬净	2（14.3）

**注** CMV：巨细胞病毒；EBV：EB病毒；G试验：（1，3）-β-D-葡聚糖试验；EORTC/MSG：欧洲癌症研究和治疗组织-感染性疾病协作组。a：15例患者可追踪到是否进行中心静脉置管；b：6例患者进行了肝脏组织病理活检；c：14例患者进行了G试验；d：14例进行了抗真菌预防治疗

二、HSC临床特征

根据EORTC/MSG标准，18例HSC患者包括确诊1例（5.6％）、临床诊断2例（11.1％）、拟诊15例（83.3％）。共有6例（33.3％）患者进行了肝脏组织病理活检，其中仅有1例检出热带念珠菌。所有的患者均进行了真菌血培养，6例（33.3％）被确诊为HSC前存在念珠菌血症，分别为白念珠菌2例、热带念珠菌4例，此6例患者血培养阳性距离诊断HSC的中位时间为28（0～76）d。其中2例被确诊为HSC前2周内出现念珠菌血症，分别为白念珠菌和热带念珠菌，血培养阳性至HSC诊断的时间分别为6 d和7 d；诊断HSC时，4例患者无相关数据，其余14例均未处于粒缺期。14例非粒缺患者中，9例（64.3％）HSC发生在粒细胞恢复后，中位发生时间为粒细胞恢复后30（5～77）d。18例患者中，4例（22.2％）患者存在口腔黏膜损伤，5例（27.8％）患者接受了胃肠外营养，1例（5.6％）患者曾在ICU接受治疗。在可追踪到的15例患者中，12例（80.0％）曾在HSC诊断前进行了中心静脉置管。除此之外，18例患者中有4例（22.2％）患者合并了巨细胞病毒血症，1例（5.6％）患者合并了EB病毒血症。

所有的患者均有持续的高热，2例（11.1％）出现腹痛。查体提示2例（11.1％）出现肝肿大，4例（22.2％）出现脾肿大。18例患者肝脏CT均提示有多发异常表现，5例（27.8％）肝内多发低密度结节，4例（22.2％）肝内多发低密度灶，5例（27.8％）肝内多发低密度影；除此之外1例（5.6％）肝脏实质不均，1例（5.6％）肝内有多发占位，1例（5.6％）有肝内多发钙化灶，1例（5.6％）仅提示有多发病变。12例（66.7％）患者脾脏CT提示有多发异常表现，其中3例（25.0％）表现为多发低密度结节，2例（16.7％）表现为多发低密度灶，4例（33.3％）表现为多发低密度影，另有1例（8.3％）表现为多发钙化灶，2例（16.7％）仅表现为脾脏多发病变。6例（33.3％）患者同时进行了超声检查，其中3例超声检查结果提示有肝脏和（或）脾脏内低密度灶、不均质改变、多发低回声区等。3例（16.7％）患者进行了MRI检查，提示肝脏和（或）脾脏的多发占位、异常信号灶、多发异常密度结节。

在18例患者中，14例（77.8％）患者进行了（1，3）-β-D-葡聚糖试验（G试验）检测，其中6例（42.9％）G试验阳性。

三、肝脾念珠菌病的治疗及结局

在本研究的18例患者中，13例（72.2％）采用了单药治疗，其中氟康唑1例（7.7％）、伏立康唑7例（53.8％）、泊沙康唑1例（7.7％）、卡泊芬净3例（23.1％）、两性霉素B 1例（7.7％）；5例（27.8％）采用联合抗真菌治疗方案，其中伏立康唑联合卡泊芬净、伏立康唑联合两性霉素B、艾沙康唑联合两性霉素B各1例，卡泊芬净联合脂质体两性霉素B 2例。

在本研究中的1例活检阳性和6例血培养阳性的患者中，仅有2例进行了药敏试验。1例未进行抗真菌预防治疗患者，药敏试验显示为白念珠菌，对伊曲康唑耐药，对伏立康唑、卡泊芬净敏感；而另1例采用氟康唑作为再次抗真菌预防治疗药物，药敏结果为热带念珠菌，对氟康唑、伏立康唑耐药，对棘白菌素类（包括阿尼芬净、卡泊芬净和米卡芬净）敏感。

在患者诊断HSC 4周时进行疗效评估显示，在可获取疗效的17例患者中，10例（58.8％）达部分缓解，4例（23.5％）疾病稳定，3例（17.6％）疾病进展。而在8周时获取到16例患者的临床缓解情况显示，完全缓解、部分缓解、疾病稳定、疾病进展的患者分别为1例（6.3％）、10例（62.5％）、3例（18.8％）、2例（12.5％）。在诊断HSC后6个月时的随访显示，18例患者中3例（16.7％）死亡，均死于血液学复发。除此之外10例（55.6％）达到部分缓解，3例（16.7％）疾病稳定。

四、HSC对后续治疗的影响

在18例患者中，3例（16.7％）在异基因造血干细胞移植后发生HSC的患者未进行后续化疗。另有15例（83.3％）在化疗后发生HSC的患者进行了后续治疗，其中7例（46.7％）在诊断HSC后继续接受了化疗；8例（53.3％）在诊断HSC后进行了异基因造血干细胞移植，包括7例半相合移植和1例全相合移植。对15例患者接受后续治疗（移植/化疗）后1个月和6个月时HSC的疾病状态进行回顾性分析，在接受后续治疗（移植/化疗）1个月时，3例（20.0％）出现疾病进展、8例（53.3％）部分缓解、4例（26.7％）疾病处于稳定状态；无患者出现复发和死亡。接受后续治疗（移植/化疗）6个月时，在可追踪疾病状态的13例患者中2例（15.4％）的HSC出现疾病进展、8例（61.5％）达到部分缓解、3例（23.1％）疾病处于稳定状态；15例患者中共有3例（20.0％）死亡，均死于血液学复发，无患者死于HSC（表2）。

**表2:** 18例肝脾念珠菌病（HSC）患者后续治疗特征

例号	性别	年龄（岁）	基础血液疾病	诊断级别	病原菌	临床特点	后续治疗方案	诊断至后续治疗时间（d）	粒细胞植活时间（d）	血小板植活时间（d）	aGVHD	cGVHD
1	女	54	AML	拟诊	热带念珠菌	持续发热	HSCT	117	13	15	是	否
2	女	24	ALL	拟诊	未检出	持续发热、腹痛	HSCT	118	12	15	否	否
3	男	33	AML	拟诊	未检出	持续发热	HSCT	98	10	12	是	否
4	男	37	AML	拟诊	热带念珠菌	持续发热、肝脾肿大	HSCT	109	22	44	是	否
5	男	36	AML	拟诊	未检出	持续发热、肝脾肿大	HSCT	98	14	71	是	否
6	男	33	AML	拟诊	未检出	持续发热、脾肿大	HSCT	31	13	17	是	是
7	男	20	MPAL	拟诊	未检出	持续发热	HSCT	135	19	36	是	否
8	男	25	ALL	拟诊	未检出	持续发热	HSCT	398	15	17	是	否
9	男	44	AML	拟诊	未检出	持续发热	化疗	10	NA	NA	NA	NA
10	男	58	AML	拟诊	未检出	持续发热	化疗	67	NA	NA	NA	NA
11	男	39	AML	临床诊断	白色念珠菌	持续发热	化疗	20	NA	NA	NA	NA
12	男	40	AML	确诊	热带念珠菌	持续发热、脾大	化疗	10	NA	NA	NA	NA
13	男	38	AML	临床诊断	热带念珠菌	持续发热	化疗	50	NA	NA	NA	NA
14	女	36	AML	拟诊	白色念珠菌	持续发热	化疗	9	NA	NA	NA	NA
15	男	45	ALL	拟诊	未检出	持续发热	化疗	10	NA	NA	NA	NA
16	男	18	ALL	拟诊	热带念珠菌	持续发热、腹痛	无	NA	16	13	否	是
17	女	25	ALL	拟诊	未检出	持续发热	无	NA	9	8	是	否
18	男	4	ALL	拟诊	未检出	持续发热	无	NA	14	16	是	否

**Table:** 

例号	aGVHD	cGVHD	CMV感染	EBV感染	后续治疗1个月HSC状态	后续治疗1个月生存状态	后续治疗2个月HSC状态	后续治疗2个月生存状态	后续治疗6个月HSC状态	后续治疗6个月生存状态
1	是	否	否	否	部分缓解	存活	部分缓解	存活	部分缓解	存活
2	否	否	是	是	部分缓解	存活	部分缓解	存活	部分缓解	存活
3	是	否	否	否	疾病稳定	存活	部分缓解	存活	部分缓解	存活
4	是	否	否	否	部分缓解	存活	完全缓解	存活	—	存活
5	是	否	否	否	疾病进展	存活	部分缓解	存活	部分缓解	存活
6	是	是	否	否	疾病稳定	存活	疾病稳定	存活	疾病稳定	死亡（复发）
7	是	否	否	否	部分缓解	存活	疾病稳定	存活	疾病稳定	死亡（复发）
8	是	否	是	否	疾病进展	存活	部分缓解	存活	部分缓解	存活
9	NA	NA	否	否	部分缓解	存活	部分缓解	存活	部分缓解	存活
10	NA	NA	否	否	部分缓解	存活	疾病进展	存活	疾病进展	存活
11	NA	NA	否	否	疾病稳定	存活	疾病稳定	存活	疾病稳定	存活
12	NA	NA	否	否	疾病稳定	存活	疾病进展	存活	疾病进展	存活
13	NA	NA	否	否	部分缓解	存活	部分缓解	存活	部分缓解	存活
14	NA	NA	否	否	部分缓解	存活	部分缓解	存活	部分缓解	存活
15	NA	NA	否	否	疾病进展	存活	—	存活	—	死亡（复发）
16	否	是	是	否	NA	NA	NA	NA	NA	NA
17	是	否	是	否	NA	NA	NA	NA	NA	NA
18	是	否	否	否	NA	NA	NA	NA	NA	NA

**注** AML：急性髓系白血病；ALL：急性淋巴细胞白血病；MPAL：混合表型急性白血病；HSCT：造血干细胞移植；aGVHD：急性移植物抗宿主病；cGVHD：慢性移植物抗宿主病；CMV：巨细胞病毒；EBV：EB病毒；NA：不适用；—：缺失

## 讨论

HSC十分罕见，预计发病率<1％[2],[5],[11]。HSC的发病机制尚不清楚。有研究认为，HSC的发生可能与中性粒细胞恢复后辅助性T细胞1/辅助性T细胞17（Th1/Th17）和辅助性T细胞2/调节性T细胞（Th2/Treg）反应下调所导致的免疫重建综合征有关[1],[12]。目前对于HSC的流行病学和临床特征了解较少，本研究对18例血液病合并HSC患者的临床特征及预后进行了描述，为国内同行更好理解HSC提供参考。

在本研究中患者的主要临床表现为持续发热、腹痛等，提示在此类患者中，尽管临床表现缺乏特异性，临床医师应考虑到HSC的可能性。组织病理活检是HSC确诊的金标准[13]，但组织病理活检的阳性率较低，在一项关于肝念珠菌感染的研究中，活检的敏感度为42％，在接受抗真菌治疗的患者中敏感度仅为30％[4]。本研究中，共有6例（33.3％）患者进行了肝脏活检，检出热带念珠菌1例，未知病原体1例，其余4例为阴性。组织病理活检阳性率可能与肝脏无菌收集受感染组织培养物的时间和采样部位有关系[13]。此外，作为一项侵入性检查手段，对于免疫功能低下的血液系统恶性肿瘤/异基因造血干细胞移植患者来说，绝大多数疑似HSC患者无法进行肝脏组织活检[14]。影像学检查是目前HSC诊断的主要手段，腹部超声可提示有肝脏和（或）脾脏内低密度灶、不均质改变、多发低回声区。过去腹部超声曾作为一种主流诊断方式，然而检出率相对较低[15]，在当前的临床工作中，在怀疑患者肝脏和（或）脾脏存在HSC时，通常会使用CT作为常规检查[15]；当CT检查不能检出但仍然存在可疑病变时，可以通过MRI检查进一步诊断。在我们的研究中，18例HSC患者均有不同程度的CT表现，且3例患者MRI检查提示肝脏和（或）脾脏的多发占位、异常信号灶、多发异常密度结节。近年来随着影像学技术的不断发展，PET-CT在HSC诊断中具有潜在的价值[13]，但并非HSC诊断的主流方法，对于其在HSC中的作用需要进一步研究。此外，甘露聚糖抗原（β-葡聚糖）的检测可能是早期诊断HSC和监测治疗反应的有用的间接标志物[16]–[17]，本研究14例进行G试验检测的患者中6例（42.9％）阳性。

HSC的病情迁延，通常需要长期抗真菌药物治疗和定期的随访，过去氟康唑是抗真菌治疗的一线药物[9]，近年来随着广泛的抗真菌预防治疗，目前美国感染病学会（IDSA）、欧洲临床微生物学和感染病学会（ESCMID）推荐棘白菌素类或两性霉素B作为一线抗真菌药物[18]–[19]。对于HSC的临床疗效评估通常依赖于临床症状和体征、影像学表现、微生物学检查等。在本研究中HSC诊断4周时的随访显示，没有患者达到完全缓解；而在HSC诊断8周时，仅有1例（6.3％）患者达到完全缓解。在诊断6个月时，有3例（16.7％）患者死于血液病的复发，而并无HSC相关的死亡。这与文献[20]报道相似。

在对HSC进行良好控制的前提下，多数指南及研究均认为HSC不影响后续化疗及造血干细胞移植患者的治疗[14]。一项14例具有侵袭性真菌感染的患者接受异基因造血干细胞移植的研究显示，所有患者均表现出植入后影像学稳定或改善，不需要与侵袭性真菌感染相关的手术，也没有发生与侵袭性真菌感染相关的死亡[14]。我们的研究同样表明HSC患者接受后续造血干细胞移植或化疗是安全可行的。

近年来随着抗真菌预防药物的广泛使用，突破性IC的比例日益升高。除了宿主免疫应答受损外，目前研究表明突破性IC主要原因之一是耐药[21]。而抗真菌药物的广泛使用被认为是念珠菌耐药的主要驱动因素[22]–[24]。中国医疗机构真菌病监测能力与管理现状报告（2020）[25]显示，白念珠菌、近平滑念珠菌对氟康唑、伏立康唑的敏感性保持在85％以上，而热带念珠菌对氟康唑、伏立康唑耐药情况较为突出，耐药率约为40％。除此之外，获得性棘白菌素耐药也有报道，特别是光滑念珠菌和热带念珠菌，既往一项抗真菌检测项目结果显示，在棘白菌素耐药的菌株中检测到FKS基因的位点突变可导致对两种和两种以上的棘白菌素耐药[26]。突破性IC的增加突出表明抗真菌药敏试验的重要性，以此对患者进行针对性的治疗；其次在临床中应监测耐药菌株以便进行及时的药物调整[21]。

总之，HSC作为血液病患者的一种少见合并症，在出现持续发热、肝脾肿大时应警惕其发生可能性；患者的抗真菌治疗时间长，在HSC有效控制后，化疗或造血干细胞移植总体上是安全的。
